# Estimated Dietary Fluoride Intake by 24-Month-Olds from Chocolate Bars, Cookies, Infant Cereals, and Chocolate Drinks in Brazil

**DOI:** 10.3390/ijerph20043175

**Published:** 2023-02-11

**Authors:** Tamara Teodoro Araujo, Samanta Mascarenhas Moraes, Thamyris de Souza Carvalho, Larissa Tercilia Grizzo, Marilia Afonso Rabelo Buzalaf

**Affiliations:** Department of Biological Sciences, Bauru School of Dentistry, University of São Paulo, Bauru 17012-901, São Paulo, Brazil

**Keywords:** fluoride intake, dental fluorosis, chocolate cookies, infant cereal, chocolate milk

## Abstract

The use of fluoride (F) in the prevention of dental caries is established. However, a high amount of F intake during tooth development can cause dental fluorosis The aim of this study was to analyze variations in F concentrations in chocolate bars (CB), chocolate cookies (CC), infant cereals (IC), and chocolate milk drinks (CD) to determine the daily intake of F from different sources by children at the age of risk for developing dental fluorosis. Distinct brands of CB, CC, IC, and CD were analyzed. Fluoride was separated by hexamethyldisiloxane-facilitated diffusion. Analysis was made in triplicate with an F ion-specific electrode. F ingestion (mg/kg body weight) was evaluated with the suggested consumption (0.05–0.07 mg/kg/day) for children aged 24 months (12 kg). The concentrations for all the analyzed products ranged from 0.025 to 1.827 µg/g F. The mean (range) F concentrations were CB= 0.210 ± 0.205 µg/g (0.073–0.698, n = 8), CC = 0.366 ± 0.416 µg/g (0.320–1.827, n = 9), IC = 0.422 ± 0.395 µg/g (0.073–1.061, n = 5), and CD = 0.169 ± 0.170 µg/mL (0.025–0.443, n = 12). The products that had the highest concentration in the categories CB, CC, IC, and CD, respectively, were Nescau-Ball (0.698 µg/g), Passatempo (1.827 µg/g), Milnutri (1.061 µg/g), and Toddynho (0.443 µg/mL). The consumption of only one unit of Toddynho (CD) is equivalent to more than 11% of the maximum suggested daily intake for a 24-month-old child (0.07 mg/kg body weight). When one product from each category is consumed together only once a day, this consumption is equivalent to approximately 24% of the suggested daily intake of fluoride for a 24-month-old child. The presence of high levels of fluoride in certain products suggests that they play a significant role in overall fluoride intake. It is crucial to closely monitor the fluoride content of food and drinks that are consumed by children who are at risk for dental fluorosis, and for product labels to clearly display the fluoride concentrations.

## 1. Introduction

Fluoride (F) has been studied since the last century as a major cariostatic agent. The use of F in the dental area has brought great positive impacts in the area of public health [1]. However, excessive ingestion of F during the period of tooth formation may lead to dental fluorosis (DF). There are several sources of F intake, such as oral hygiene products, water, and food [2,3,4,5].

Studies have shown that ages between 6 and 9 months present great potential for the development of DF in the first dentition, with a higher prevalence in the first and second deciduous molars [6,7,8]. On the other hand, up to the ages of 6 to 8 years, all permanent teeth have an increased risk of being affected by DF [9,10,11]. However, for the maxillary central incisors that are of greatest cosmetic importance, the critical period of susceptibility to DF comprises the first 3 years of life [12]. Recent studies have shown an increase in the prevalence of DF, both in the primary [7,8,13] and in the permanent [9,10,14] dentitions. Therefore, it is extremely important to assess children’s sources of F intake individually, as total F intake impacts the development of DF [5,15,16].

The ‘optimal’ level of F intake that provides maximum protection against caries with minimum risk of provoking DF is not known so far [16]. A plethora of factors interfere in the metabolism of F, affecting the balance between F intake and retention by the organism and, therefore, modifying the risk of developing DF [3]. This might be responsible for the current unavailability of a Dietary Reference Value for F [17]. The Institute of Medicine of the USA (IOM) elaborated on what would be the adequate intakes (AIs), based on the minimization of caries and the reduction of the adverse effects of F on the health of individuals as a reference. AI values were 0.01 mg/day for infants from birth to 6 months and 0.05 mg/kg of body weight/day for children older than 6 months and adults. The Institute even suggested a daily intake of 0.1 mg/kg of body weight/day as the upper limit of F for infants and children up to 8 years of age [18]. Due to the lack of evidence on the ‘optimal’ level of F intake, the range of 0.05–0.07 mg/kg body weight/day was empirically established and is still employed [19].

In addition, studies have indicated changes in children’s eating patterns over the last three decades [3,20]. This may have been attributed to the consumption of different types of food, perhaps due to more industrialized options, as well as to different methods of analyzing the F that is present in the products that are more commonly available in the market [21,22]. Currently, it is observed that in different age groups, children are exposed to distinct sources of F, but there are only a few studies that report the risk-benefit relationship of F intake through consumption of foods and beverages [9,23]. Moreover, the F concentration is not stated on the products’ labels, which means that it is necessary to analyze their F content in order to provide information to parents of children at the age of risk to DF on products that could potentially be important contributors for the total daily F intake of their children.

Thus, in the present study, the amount of F present in infant cereals, chocolate drinks, chocolate bars, and chocolate biscuits that are commonly available in Brazil was evaluated. The concentrations found were employed to estimate the contribution of these foods and beverages for the total daily F intake of 24-month-old children (~12 kg), who are subject to a greater risk of developing DF fluorosis in the upper central incisors.

## 2. Materials and Methods

A total of five samples of infant cereal products (IC), twelve chocolate milk products (CD), eight chocolate bars (CB), and nine chocolate cookies (CC) of different brands (Table 1) were purchased in the market of Bauru and Sorocaba, São Paulo, Brazil, with 3 batches of each product. The products were chosen because they are very popular among babies and young children, have attractive packaging for this public, and are available in all Brazilian states. Moreover, some of these products have been shown in previous studies [13,20,24,25,26,27] to have high F content. In addition, some products had a lower sample size due to the low variability of brands in the market, such as infant cereals. Foodstuff type, brand name, manufacturer’s name, and place of production were indicated for each studied product (Table 1).

### Preparation and Fluoride Analysis

The packages were only opened on the day of the analyses. The amounts of ICs and CDs weighed 0.4 g and 0.4 mL, respectively. CBs were initially frozen and then grated and then weighed (0.2 g). For the CCs, the fillings were separated from the wafers, and both were macerated. After this preparation, 0.3 g was used for F analysis.

All the samples were placed in plastic Petri dishes. Each item was weighed and analyzed in triplicate. All the samples were diluted in deionized water (Purelab Option-Q, Veolia Water Technologies, Buenos Aires, Argentina). Knowing the exact amounts of ICs, CDs, CBs, and CCs it was possible to calculate the F content of the original, dry products.

Fluoride determinations were performed as previously described [28], after overnight hexamethyldisiloxane (HMDS)-facilitated diffusion, using the F ion-specific electrode (model 9409, Orion Research, Cambridge, MA, USA) coupled to a calomel reference electrode (Accumet, #13-620-79). Standards containing 0.25, 0.5, 1.0, 5.0, 10.0, and 50.0 nM F were prepared and diffused along with the samples to be analyzed. The millivoltage readings were converted to µg F using a standard curve with a coefficient of r^2^ ≥ 0.99. All samples were analyzed in triplicate.

## 3. Results

Table 1 shows the name, manufacturer, and production site of the infant foods and beverages analyzed.

The results that were obtained for the ICs are shown in the Table 2, as well as the brands, manufacturers, and the mean F concentrations, expressed in µg/g. The ICs group had a mean [F] ± SD (amplitude; µg/g) of 0.422 µg/g ± 0.395 (0.073–1.061, n = 5). The suggested range of F intake is 0.05 to 0.07 mg F/kg body weight/day. Therefore, the IC results showed that for the two brands with the highest concentrations [Milnutri Arroz e Aveia (1.061 µg/g—Danone) and Neston Vitamina (0.496 µg/g—Nestlé)], 30 g is equivalent to almost 4%–5% and 2%–3%, of the maximum daily F intake for a 24-month child (12 kg), respectively, based on the suggested consumption.

The mean [F] SD and amplitude (unit µg/g) of the chocolate drinks (CD) were 0.169 µg/g ± 0.175 (0.025–0.443, n = 12), shown in Table 3. In this group we found a slightly high F concentration in two brands: Nescau Zero Lactose (0.425 µg/g—Nestle) and Toddynho (0.443 µg/g—Pepsico). The consumption of one unit (200 mL) of Nescau Zero Lactose or Toddynho represents 10–14% and 11–15% of the daily range of F intake for a 24-month-old child (12 kg), respectively.

Most of the products from the chocolate bars (CB) category in the Table 4 had low F concentrations, except for the Nescau Ball group (0.698 µg/g—Nestle), which in terms of the range of daily F intake represents almost 3–5% of the suggested consumption (40 g). The mean [F] SD and amplitude (unit µg/g) of CB was 0.210 µg/g ± 0.192 (0.073–0.698, n = 8).

The chocolate cookies (CC) showed in the Table 5 only two brands with lower F concentration. The mean [F] SD and amplitude of the CC group (unit µg/g) was 0.849 ± 0.392 (0.320–1.827, n = 9). The only brand with lower F concentration was Nikito chocolate (0.320 µg/g—Vitarella). On the other hand, all other brands showed higher F concentrations. The product with the highest F concentration was Passatempo chocolate (1.827 µg/g—Nestlé), which represents 7–9% of the range of daily F intake for a 24-month child (12 kg).

## 4. Discussion

Even though F is not generally regarded as an essential element, it has an important role on the mineralization of hard tissues and on caries control, and is included in the list of ultratrace elements (an element with an established or estimated requirement, generally indicated in µg/day for humans) [29]. There are three main ways to deliver F to control caries: community-based methods (such as fluoridated water, salt, and milk), professionally administered methods (such as F gels and varnishes), and self-administered methods (such as toothpastes and mouthwashes). The best caries-preventive effect of F occurs when the ion is present, even in low concentrations, in the fluid phases of the oral environment surrounding the teeth [30,31]. The mode-of-action of F is essentially post-eruptive and can be attributed mainly to its influence on the de- and re-mineralization kinetics of dental hard tissues [1,32,33].

Although evidence on the dental effects of topical F is widely accepted, the risk-benefit ratio of systemic exposure to F ingestion is a complex scenario, as excessive intake during tooth development can increase the risk of DF [34]. Several studies have reported an increase in the occurrence of this dental condition in both primary [7,8,13] and permanent dentitions [9,10,14,35]. Studies on the timing of F intake and fluorosis have focused on the most aesthetically important teeth, the maxillary central incisors [10,11,12,36,37]. In a meta-analysis aimed at defining a “risk period” for the development of fluorosis in upper permanent central incisors, Bardsen [11] concluded that the duration of exposure to F during the process of amelogenesis is a significant predictor of risk for fluorosis. Additionally, it was determined that it is challenging to identify specific periods as being more hazardous. Evans et al. [36] found that males are most susceptible to fluorosis of maxillary central incisors around 15–24 months, and females around 21–30 months. In addition, children who were exposed to high levels of F during their first and second years of life had an increased risk of developing DF on their maxillary and mandibular central incisors, as well as their first molars [12,37].

Recent trends in infant feeding habits and parenting practices have led to increased consumption of processed foods that may contain high levels of F [38,39], which may contribute to the development of DF. Therefore, it is particularly important to analyze the F content of foods consumed by children more often. Due to this, we focused on choosing these popular Brazilian products to evaluate their F concentration.

Conducting studies on food intake in 24-month-olds is crucial, as it has been shown that at this age, dietary sources contribute to 53% of their F intake [5]. Research has suggested that a total F intake of 0.05–0.07 mg/kg body weight per day in children can provide dental health benefits. However, it is important to ensure that F intake does not exceed this level to minimize the risk of DF, particularly during the process of enamel formation [19]. Considering this range, the total F intake for a 24-month-old child weighing 12 kg varies from 0.6 to 0.84 mg F per day. Due to the wide variety in F-containing commercial products for 24-month-old children, the selected products were based on previous studies in which similar products were shown to have a high F content [20,24,25,26,27]. Moreover, some of them were chosen because they are in attractive and colorful packaging with children’s characters. To make easier the analytical procedures and discussion of the results, the products were divided in groups: infant cereals, chocolate drinks, chocolate bars, and chocolate cookies.

A large variation in F concentration is observed in the literature for infant cereals. A study by Dabeka et al. [40] analyzed 334 commercial infant foods in Canada and found that infant cereals had a range of F concentrations between 1.24 and 4.89 µg/g. Additionally, Buzalaf et al. [24] found that F concentrations in infant cereals ranged between 0.43 and 6.64 µg/g, and observed consistency in values that were obtained from the same product manufactured on different dates. In 2004, Buzalaf et al. [26] found higher F concentrations (between 2.11 and 7.84 µg/g) than in 2002. Another study reported similar values (4–6 µg/g) [39]. All the cereals that wer analyzed in this study, except one, had low F concentrations (between 0.073 and 1.061 µg/g). This is in agreement with Vlachou et al. [41] and Wiatrowski et al. [42] (0.01–0.31 µg/g; 0.63–1.17 µg/g), respectively. This difference in F values found in cereals may be related to the production of these foods with the use of fluoridated water with different concentrations of this chemical element.

The F concentrations in chocolate drinks were found to vary greatly among the brands that were analyzed, ranging from 0.025 to 0.443 µg/mL. There were two brands, Nescau Zero Lactose (Nestlé^®^) and Toddynho^®^ (Pepsico) that were found to have F concentrations above 0.4 µg/mL, with measurements of 0.425 µg/mL and 0.443 µg/mL, respectively. Despite previous studies of our group already identifying high levels of F in chocolate milk, the source of F could not be found [20,24,26]. Some brands of chocolate milk were found to have fluoride levels that exceeded the threshold dose associated with the development of dental issues, results that are consistent with the literature [20,24,26]. One unit of Toddynho^®^ (Pepsico) or Nescau Zero Lactose (Nestlé^®^) can reach 15% or 14% of the maximum daily F intake for a 24-month child (12 kg), respectively. Thus, it is of great importance that there is monitoring of the amount of intake of these chocolate drinks by parents.

Most of the products from chocolate bars had low F concentrations (the lowest was Tortuguita brigadeiro with 0.073 µg/g), except for the Nescau Ball group (0.698 µg/g—Nestlé). These findings are consistent with the literature that reports an F concentration ranging from 0.07 to 1.60 µg/g [25]. A packet of Nescau ball (75g) is equivalent to 6–9% of total daily F intake for a 24-month child (12 kg). The chocolate cookies showed only two brands with lower F concentrations. On the other hand, all the other brands showed higher F concentrations. Passatempo chocolate had the highest F concentration (1.827 µg/g—Nestlé). Even though high concentrations are found, they are still below those that are reported in the literature, 6.9–13.7 µg/g [26] and 7.1 µg/g [25]. When a 2-year-old child weighing 12 kg consumes only three units of Passatempo chocolate once a day, it accounts for up to 7% of their maximum recommended daily F intake of 0.07 mg/kg. It is interesting to mention that three units of Passatempo, according to the manufacturer, contains 19 g of carbohydrates. This covers only around 10% of the daily carbohydrates demand of a 2-year-old child, considering a diet of 1300 kcal, which means that it is likely that one child consumes more than three units on a single day.

Many studies measure the amount of F in foods [15,20,24,25,27], but it is important to also consider how much of that F is absorbed. Since it can be difficult to conduct human bioavailability studies, determining the F that can dissolve in the gastric juice is a key area of focus. Buzalaf et al., in 2004, found that all of the F that was present in cereals was soluble (SF). However, in chocolate-flavored milk, only about half of the total F (TF) was SF. Similarly, for biscuits, only around 20% of TF was SF. This may be because high levels of calcium in milk and calcium-rich biscuits, as indicated on their labels, can bind to F and make it less available for absorption. The study suggested that certain cereals, beverages, and biscuits may be significant sources of daily F intake [26]. Surprisingly, among the chocolate cookies, Passatempo that had the highest concentration of F is also the cookie that has the highest amount of calcium according to the nutritional information on the label.

In another study, Trautner and Einwag [43] suggested that the formation of calcium salts and entrapment of F in the coagulation products of milk can reduce F bioavailability. They also propose that by prolonging the stay of chyme after consuming food, increases the bioavailability, since the digestion processes can liberate F from bound forms and coagulation products [43]. In addition, Nopakun et al. [44] reported that only a quarter of F absorption occurs in the stomach, with the remainder taking place in the small intestine. This suggests that the daily F intake that is estimated through the measurement of hydrochloric acid (HCl) SF may be an underestimation. In addition, there is some evidence that lower intake of calcium and vitamin D in lower socioeconomic status adolescents increases the bioavailability of F and the risk of severe fluorosis [45]. In fact, the bioavailability of F in vivo is complex and definitive conclusions can only be drawn through in vivo studies that are conducted on human subjects.

It is possible that the high F concentrations that were identified in these products may represent substantial contributions to the overall daily F intake. For a 2-year-old, 24% of the maximum suggested recommended daily F intake is 0.07 mg F/kg/day. When one portion of the product with the highest F content from each category is consumed only once a day, the total amount of F that is ingested reaches about 24% the upper limit (0.07 mgF/kg/day) of the F intake that is regarded as “optimal” to prevent caries with minimum risk of causing DF [3]. By identifying potential sources of high F intake, recommendations may be to reduce consumption of these sources in patients who may be at risk for DF.

The wide range of F content within food and beverage groups verified in the UK fluoride database [34], in several Brazilian studies [20,24,25,26,27] and in a recent study in the US [21] accentuates the need for comprehensive F labeling of foods and beverages, especially those that are frequently consumed by infants and young children. Monitoring F intake can be challenging and labor-intensive, requiring the assessment of F ingestion from both diet and toothpaste. A study that was conducted in the US reported that some of the analyzed bottled waters that were intended for infants did not meet the American Dental Association’s (ADA) recommendation to prevent fluorosis [46]. Recently, the US Food and Drug Administration issued mandatory identification of bottled water containing F, as well as amended the allowed level for F in bottled water to which F is added to 0.7 mg/L [47]. In Europe, fewer than 26% of the brands of bottled water label their F content [47]. In Brazil, as well as in Europe, manufacturers are not required to include information about the F content on food and beverage labels [34,48]. Control of F intake, however, would be facilitated by the labeling of F content on food and drink products [34]. The identification of potential sources of excessive F intake allows the formulation of appropriate recommendations, which may include the reduction of consumption of these sources by individuals who are at risk for DF.

## 5. Conclusions

The high concentrations of fluoride that are found in some products in the present study highlight the importance of measuring the F content of foods and beverages that are consumed by young children. It is imperative to consider these products as potentially significant contributors to overall F intake. In addition, it is recommended that the F concentrations of these products be clearly stated on their labels for consumer awareness and informed decision-making.

## Figures and Tables

**Table 1:** Name, manufacturer, and production site of the infant cereals, chocolate drinks, chocolate bars, and chocolate cookies that were analyzed.

Foodstuff Type	Product Name	Manufacturer	Production Site
**Infant Cereals**	Farinha Lactea	Nestlé	Feira de Santana—BA
	Farinha Lactea	Piracanjuba	Paulista—PE
	Milnutri Arroz e Aveia	Danone	Colombo—PR
	Mucilon	Nestlé	São José do Rio Pardo—SP
	Neston Vitamina	Nestlé	São José do Rio Pardo—SP
**Chocolate Drink**	Danette	Danone	Patrocínio Paulista—SP
	Italakinho chocolate	Italac	Tapejara—RS
	Itambé Kids	Itambé	Pará de Minas—MG
	Itambé Kids Zero lactose	Itambé	Pará de Minas—MG
	Mocoquinha	Mococa	Cerqueira Cesar—SP
	Nescau	Nestlé	Feira de Santana—BA
	Nescau light	Nestlé	Feira de Santana—BA
	Nescau zero lactose	Nestlé	Araçatuba—SP
	Pirakids	Piracanjuba	Bela Vista de Goias—GO
	Pirakids Zero lactose	Piracanjuba	Bela Vista de Goias—GO
	Toddyinho	Pepsico	Sorocaba—SP
	Toddyinho Levinho	Pepsico	Feira de Santana—BA
**Chocolate Bars**	Baton	Garoto	Caçapava—SP
	Bib’s ao leite	Neugebawer	Arroio do Meio—RS
	Disquete	Dori	Marília—SP
	Kinder ao leite barra	Ferrero	Poços de Calda—MG
	Lolo	Nestlé	Caçapava—SP
	M & M´s	Master foods	Guararema—SP
	Nescau Ball	Nestlé	Marília—SP
	Tortuguita brigadeiro	Arcor	Bragança Paulista—SP
**Chocolate Cookies**	Bauducco reacheados	Bauducco	Extrema—MG
	Bono	Nestlé	Marília—SP
	Garoto Recheado	Garoto	Marília—SP
	Nestlé Classic	Nestlé	Marília—SP
	Nikito chocolate	Vitarella	Eusébio—CE
	Passatempo chocolate	Nestlé	Marília—SP
	Plug@dos chocolate suíço	Adria	Lençois Paulista—SP
	Tortuguita brigadeiro	Arcor	Campinas—SP
	Trakinas chocolate	Mondelez	Vitória de Santo Antão—PE

**Table 2:** Mean fluoride content of the different types of infant cereals that were analyzed.

Foodstuff Type	Brand	Manufacturer	[F] (µg/g)	Mean (µg/g) ± SD
**Infant** **Cereals**	Farinha Lactea	Nestlé	0.131	
	Farinha Lactea	Piracanjuba	0.349	
	Milnutri Arroz e Aveia	Danone	1.061	0.422 ± 0.395
	Mucilon	Nestlé	0.073	
	Neston Vitamina	Nestlé	0.496	

**Table 3:** Mean of fluoride content of the different types of chocolate drinks that were analyzed.

Foodstuff Type	Brand	Manufacturer	[F] (µg/g)	Mean (µg/g) ± SD
**Chocolate** **Drinks**	Danette	Danone	0.032	0.169 ± 0.175
	Italakinho chocolate	Italac	0.024	
	Itambé Kids	Itambé	0.037	
	Itambé Kids Zero lactose	Itambé	0.098	
	Mocoquinha	Mococa	0.025	
	Nescau	Nestlé	0.380	
	Nescau light	Nestlé	0.351	
	Nescau zero lactose	Nestlé	0.425	
	Pirakids	Piracanjuba	0.027	
	Pirakids Zero lactose	Piracanjuba	0.045	
	Toddyinho	Pepsico	0.443	
	Toddyinho Levinho	Pepsico	0.137	

**Table 4:** Mean of fluoride content of the different types of chocolate bars that were analyzed.

Foodstuff Type	Brand	Manufacturer	[F] (µg/g)	Mean (µg/g) ± SD
**Chocolate** **Bars**	Baton	Garoto	0.073	
	Bib´s ao leite	Neugebawer	0.212	
	Disquete	Dori	0.207	
	Kinder ao leite barra	Ferrero	0.167	0.210 ± 0.192
	Lolo	Nestlé	0.098	
	M & M´s	Master foods	0.154	
	Nescau Ball	Nestlé	0.698	
	Tortuguita brigadeiro	Arcor	0.072	

**Table 5:** Mean of fluoride content of the different types of chocolate cookies that were analyzed.

Foodstuff Type	Brand	Manufacturer	[F] (µg/g)	Mean (µg/g) ± SD
**Chocolate** **Cookies**	Bauducco recheados	Bauducco	0.643	
	Bono	Nestlé	0.842	
	Garoto Recheado	Garoto	0.845	
	Nestlé Classic	Nestlé	0.899	
	Nikito chocolate	Vitarella	0.320	0.849 ± 0.392
	Passatempo chocolate	Nestlé	1.827	
	Plug@dos chocolate suíço	Adria	0.711	
	Tortuguita brigadeiro	Arcor	0.978	
	Trakinas chocolate	Mondelez	0.580	

## Data Availability

All data underlying the results are available as part of the article and no additional source data are required.
